# Molecular Mechanisms Underpinning Microparticle-Mediated Cellular Injury in Cardiovascular Complications Associated with Diabetes

**DOI:** 10.1155/2019/6475187

**Published:** 2019-02-19

**Authors:** Tarek Benameur, Aisha Osman, Aijaz Parray, Ali Ait Hssain, Shankar Munusamy, Abdelali Agouni

**Affiliations:** ^1^College of Medicine, King Faisal University, P.O. Box 400, Al-Ahsa, Saudi Arabia; ^2^Department of Pharmaceutical Sciences, College of Pharmacy, Qatar University, P.O. Box 2713, Doha, Qatar; ^3^The Stroke Program, The Neuroscience Institute, Hamad Medical Corporation, P.O. Box 3050, Doha, Qatar; ^4^Medical Intensive Care Unit, ECMO Team, Hamad Medical Corporation, P.O. Box 3050, Doha, Qatar; ^5^Department of Pharmaceutical and Administrative Sciences, College of Pharmacy and Health Sciences, Drake University, Des Moines, IA 50311, USA

## Abstract

Microparticles (MPs) are small vesicles shed from the cytoplasmic membrane of healthy, activated, or apoptotic cells. MPs are very heterogeneous in size (100–1,000 nm), and they harbor proteins and surface antigens specific to cells they originate from. Virtually, all cells can shed MPs, and therefore, they can be found in all body fluids, but also entrapped in tissues. Of interest and because of their easy detection using a variety of techniques, circulating MPs were recognized as biomarkers for cell activation. MPs were also found to mediate critical actions in intercellular communication and transmitting biological messages by acting as paracrine vehicles. High plasma numbers of MPs were reported in many cardiovascular and metabolic disturbances that are closely associated with insulin resistance and low-grade inflammation and have been linked to adverse actions on cardiovascular function. This review highlights the involvement of MPs in cardiovascular complications associated with diabetes and discusses the molecular mechanisms that underpin the pathophysiological role of MPs in the onset and progression of cellular injury in diabetes.

## 1. Introduction

Microparticles (MPs) are membrane-shed vesicles, ranging in size between 100 nm and 1,000 nm, that are released from the cytoplasmic membrane of activated and/or apoptotic cells. For many decades, MPs were considered inert cell debris or platelet dust derived from platelets which are rich in phospholipids and endowed of procoagulant capacity [1]. Later, the development of methods of genetic and protein profiling showed that MPs could transport cargo content including secretable and nonsecretable biological molecules such as active lipids and nucleic acids, such as coding (mRNA) and noncoding (e.g., microRNA and long noncoding RNA) RNA and DNA, in addition to membrane and cytosolic proteins to target cells [2] and are therefore recognized today as true vectors of intercellular communication and mediators of a variety of biological messages.

MPs are involved in the regulation of molecular processes within the emitting cell itself or other distant cells. Cells may export into the extracellular environment certain subcellular organelles and macromolecules or genetic material (e.g., mRNA or microRNA) because of their role in controlling certain cell functions inside the cell or because they are directly involved in the control of the process of MP shedding and release. The elimination of these molecules entrapped within MPs may alter the properties of the parent cells such as the modulation of intracellular levels of some specific microRNAs or regulatory signaling molecules and second messengers [3].

The release of MPs to the extracellular environment will bring them in contact with neighboring cells, or if they reach systemic circulation, MPs can interact with cells of different types at remote sites within the body. MPs can interact with target cells in multiple ways ranging from a ligand-receptor type of interaction to their surface antigens, through membrane fusion with target cell or internalization which allows for dumping of MP cargo content inside the target cell [3]. Target cells, if they did not degrade MP content or eliminate it outside of the cell inside new vesicles, may respond to signaling molecules brought by MPs which eventually can alter cellular functions and responses within the recipient cell [4].

The shedding of MPs from cells is a natural mechanism, and virtually, any cell in the body is capable of releasing MPs into the extracellular environment. However, the cellular mechanisms governing the shedding of MPs are not fully elucidated. Many studies have reported that MPs are present in various body fluids and solid tissues from both healthy humans and animal models; however, the number of these MPs was found to be increased in pathological states and may constitute therefore good biomarkers for the prognosis and diagnosis of multiple pathologies [5]. In relation to cardiovascular diseases and complications, MPs from different cellular origins were reported to be increased in the blood of patients including those derived from circulating cells such as platelets, leukocytes, red cells, endothelial cells, and smooth muscle cells; however, much of circulating MPs detected were from platelet origin [5].

The current review article focuses on the involvement of MPs in diabetes-induced complications. Furthermore, it discusses the molecular mechanisms that underpin the pathophysiological role of MPs in the induction and progression of cellular injury associated with diabetes.

## 2. Differences between MPs and Other Extracellular Vesicles and Mechanisms of Formation

MPs are not the only vesicles of cell origin that can be found in body fluids; other types of extracellular vesicles are also documented such as exosomes and apoptotic bodies. However, the mechanisms controlling the liberation of these different vesicles are not the same (Table 1).

### 2.1. Formation and Release of MPs

MPs are directly released from the cytoplasmic membrane of cells. They are very heterogeneous in size (100–1,000 nm) and content but contain cytosolic cargo. The mechanisms of membrane remodeling implicated in the formation of MPs are not completely understood; however, much evidence indicates that the release of MPs happens after the activation of cells either chemically or physically or when cells undergo apoptosis. The number of MPs shed, and their qualitative content was reported to vary according to the types of stimuli at the origin of MP shedding [6, 7].

The release of MPs from cell membrane formation requires membrane remodeling following cell stimulation and the activation of cascades of intracellular signals. Bleb formation from plasma membranes, an indicator of MPs shedding, was shown to take place when an influx of calcium inside the cell occurs following cell activation. Following this intracellular calcium surge, cell blebbing is allowed via the activity of cysteine protease, *μ* calpain, which leads to the breakdown of cytoskeleton constituents, talin, and *α*-actinin [8]. In platelets, it has been reported that *μ* calpain inhibition prevented MP shedding and that *μ* calpain activation was mediated through an elevation of cyclic AMP (cAMP) levels and the subsequent activation of protein kinase A (PKA) [9].

It is in general well agreed that MPs are shed when the asymmetry of membrane phospholipid distribution between the inner and outer layers is disrupted or lost. In a basal condition, the negatively charged aminophospholipid, phosphatidylserine (PS), is essentially located in the inner leaflet of the cytoplasmic membrane. However, during cell activation or apoptosis, a prominent change in membrane asymmetry is the externalization of PS to the outer leaflet. The externalization of PS is an early indicator of the process of MP shedding. Because of its negative charge, PS externalization creates an excess of negative charges at the cell surface and imbalances the molecular masses of the two leaflets, which in turn could contribute to membrane destabilization and the formation of blebs. Maintaining membrane phospholipid asymmetry is pivotal for the proper function of cell membrane.

Loss or disruption of membrane asymmetry is tightly linked to cell activation and is associated with various disease states. Phospholipid asymmetry is maintained by the selective synthesis of specific phospholipids on each side of a cell membrane. Several proteins are involved in maintaining lipid asymmetry either by persevering or by disrupting the gradient of lipid distribution between the inner and out leaflets. There are three main classes of these protein: (i) flippase, directed toward the cytosol and ATP-dependent transporters; (ii) floppases, directed toward the extracellular environment and ATP-dependent transporters; and (iii) scramblases, bidirectional and ATP-independent transporters. Scramblases allow for a random distribution of lipids between the membrane bilayers. The flippases are very selective for PS, and their action is responsible for maintaining this phospholipid mainly sequestered at the inner leaflet of the cell membrane [10]. A sustained increase in intracellular calcium following cell activation or apoptosis inhibits flippases and therefore contributes to surface exposure of PS because of the overwhelming action of floppases [11, 12]. The disruption of cell membrane asymmetry and changes in masses of the two leaflets in addition to the calcium-induced proteolytic degradation of cytoskeleton caused by the activation of *μ* calpains lead to the formation of membrane blebs and MP release [12].

MPs exposing PS at their surface provide sites for the assembly of coagulation factors and participate thus to the process of hemostasis. Platelets have a very high rate of scrambling of membrane lipids and thus can rapidly generate MPs exposing PS which contributes in preventing bleeding; however, an excessive shedding of MPs may cause thrombotic complications [12]. Sinauridze et al. [13] reported that one single platelet-derived MP had nearly the same procoagulant activity as one activated platelet despite the surface area was 2 orders of magnitude smaller suggesting that platelet-derived MPs have a procoagulant surface that is 50- to 100-fold more potent than activated platelets [13].

However, not all MPs appear to express PS at their surface. Connor et al. [14] demonstrated that in nonstimulated plasma that is poor in platelets, about 80% of platelet-shed MPs were unable to interact with annexin V. They also reported that the fraction of MPs that successfully interacted with annexin V was dependent upon the nature of the stimulus used. Naturally stimulating molecules such as collagen produced less MPs positively labeled with annexin V as compared to nonphysiological agonists such as calcium ionophore [14]. Furthermore, in our own hands, we observed that MPs exposing PS (positive for annexin V) represented, at the most, 50% of total MPs detected in the plasma from patients with metabolic syndrome, septic shock, and sleep apnea for instance [15–18]. Therefore, it is reasonable to suggest that we still lack some understating about the significance of shedding of these MPs that do not expose PS at their surface and that other cellular mechanisms are involved in their biogenesis, which warrants further research.

Other molecular events were identified to bridge the link between the sustained increase in intracellular calcium following activation and externalization of PS and MP shedding and release from cell membranes. It has been proposed that externalization of PS is related to the entry of calcium through membrane calcium channels, which follows depletion of intracellular calcium stores; this phenomenon is known as capacitative or store-operated calcium entry (SOCE), and it has been shown that this event together with the externalization of PS is partly regulated by actin cytoskeleton [19]. The small GTPase Rho A, which interacts with the extracellular signal-regulated kinase (ERK) pathway, was found to regulate SOCE and the surface externalization of PS through the rearrangement of actin cytoskeleton [20]. The phosphorylation of myosin light chain (MLC) causes actin/myosin-mediated contractile tension, leading thus to an increase in the pressure on the cytoplasm structure, which eventually causes membrane bleb formation. The phosphorylation of MLC was shown to be mediated, during apoptosis, by the serine/threonine kinase Rho-associated protein kinase- (ROCK-) I, one of the effectors downstream of the small GTPase Rho. ROCK-I was found be activated in apoptosis by proteolytic cleavage by caspase 3, and the active kinase can then simultaneously phosphorylate and hence activate MLC, then phosphorylate and thus inactivate MLC phosphatase [12, 21, 22].

### 2.2. Formation and Release of Exosomes and Apoptotic Bodies

Another example of extracellular vesicles found in body fluids including systemic circulation is exosomes that have a size ranging between 40 and 150 nm [23–25]. Exosomes are released upon the fusion of multivesicular bodies with cell membrane. Their content is very distinct from MPs owing to their specific mode of formation. The formation of exosomes takes place within endosomes by introversion of the delineating membranes causing the generation of multivesicular bodies. Subsequently, these multivesicular bodies merge with the cytoplasmic membrane releasing thus exosomes outside of the cell [26].

The detection of exosomes is supported by both structural and molecular features. Because of their small size, exosomes can be collected following high-speed centrifugations above 100,000 *g*. Exhaustive proteomic profiling of MPs and exosomes derived from many cell types found that exosomes are exclusively enriched in some stereotypic protein markers such as TSG101, cell death 6-interacting protein (PDCD6IP), and tetraspanin 30 (CD63) while MPs do not express these markers [27, 28].

Cells undergoing apoptosis can release larger vesicles referred to as apoptotic bodies with sizes larger than 1,000 nm which are generated by the uncoordinated blebbing of cytoplasmic membrane and can carry nuclear parts and proteins. Under certain conditions, the number of apoptotic bodies exceeds those of exosomes and MPs and their content can differ between various body fluids [2].

These various types of membrane vesicles found in the extracellular environments can be discriminated according to multiple structural and physical properties. Sequential rounds of high-speed centrifugations are the most effective method to isolate cells, debris, apoptotic bodies, MPs, and exosomes. MPs are collected from the supernatant resulting from the elimination of cell debris by additional cycles of centrifugation with a speed ranging between 10,000 and 21,000 *g* whereas the isolation of exosomes requires ultracentrifugation speeds above 100,000 *g* (Table 1). It is possible to discriminate between apoptotic bodies, MPs, and exosomes by electron microscopy or flow cytometry, although owing to their detection limits, flow cytometers are believed not be optimal for the detection of exosomes because of their small size.

## 3. Composition of MPs

### 3.1. Protein Content of MPs

MP content mirrors the remodeling and rearrangement of the cytoplasmic membrane of the parent cell that is shedding them. Several studies including those involving molecular profiling methods such as proteomic techniques revealed that protein cargo content of MPs does not only depend on the nature of parent cells but also depend on the stimulating conditions at the origin of MP generation [29, 30]. It has been reported previously for example that MPs engineered *in vitro* from T lymphocytes using different stimuli carry key differences in their cargo protein content. It was found that MPs, generated from T lymphocytes activated with phytohemagglutinin (PHA) for 72 hours and then induced in apoptosis with additional stimulation for further 24 hours with phorbol-12-myristate-13 (PMA) and actinomycin D, expressed the morphogen sonic hedgehog (Shh) [7]; however, apoptotic T lymphocytes (stimulated with actinomycin D only for 24 hours) generated MPs which were deficient in Shh [6]. It has been observed that only the treatments of T lymphocytes with PHA, PMA, and actinomycin D or with PHA/actinomycin D were able to generate MPs carrying Shh [31]. However, the mechanisms behind this differential expression in protein content are not yet clear.

The differential expression of Shh translated into differences in the biological messages carried out by MPs generated from T lymphocytes. T lymphocyte MPs harboring Shh induced nitric oxide (NO) release from endothelial cells and reversed endothelial dysfunction causing ischemia/reperfusion of the left descending anterior coronary artery in mice [7] and improved endothelial dysfunction in a mouse model of angiotensin II-induced hypertension [32]. However, T lymphocyte MPs deficient for Shh reduced NO bioavailability in endothelial cells and caused endothelial dysfunction in mouse aortas [6]. MPs carrying Shh were found to activate the Shh signaling pathway in target cells [33].

Proteomic analysis of MPs derived from control and *β*-cells of islets of Langerhans stimulated with cytokines for 24 hours showed that several proteins were differentially expressed by the two populations of MPs. Multiple members of the tumor necrosis factor (TNF) transduction pathway were upregulated in MPs obtained from cells stimulated with cytokines, such as the TNF receptor superfamily member 1A, TNF*α*-induced protein 3, and TNF-interacting kinase receptor-interacting serine-threonine kinase 1 [34].

Xu et al. [35] performed a proteomic analysis of circulating MPs from newly diagnosed type-2 diabetics and healthy volunteers. The authors found 46 proteins that were differentially expressed by MPs between diabetic patients and healthy volunteers. Of these proteins, 20 proteins were increased in diabetic MPs and 17 others were only found in MPs from healthy subjects. Of interest, it was found that diabetic MPs expressed significantly higher levels of Ras-related protein Rap-1b (RAP1B), CD9, and integrin alpha-IIb (ITGA2B, CD41) [35]. All these molecules play a role in hemostasis and platelet activation and aggregation; however, the increase in CD41 expression is not surprising because this is an essential platelet glycoprotein and that platelet-derived MPs represent over 80% of all circulating MPs [5]. Xu and colleagues [35] also reported that the expression of S100A8 and S100A9 was significantly elevated in MPs from diabetic patients [35]. A previous proteomic study also found that S100A8 and S100A9 were highly increased in chronic diabetic foot exudates compared to exudates from split-skin donor sites of burn victims who are otherwise healthy [36]. Both S100A8 and S100A9 are inflammatory mediators, which can bind to advanced glycation end-products (AGEs) receptors and toll-like receptors (TLRs) to let go inflammatory cytokines [37]. The genetic deletion of S1009A in atherosclerosis-prone Apo E-deficient mice reduced atherogenesis [38]. These findings support reports indicating that MPs from diabetic patients, especially those from platelets, have a stronger procoagulant potential which contributes to the hypercoagulability in these patients and strengthens the evidence for a potential role of MPs in vascular pathogenesis in diabetes.

Because they originate from the cell membrane, MPs are enriched in membrane proteins from the parent cell such as integrins, glycoprotein Ib (GPIb), and P-selectin [2]. These surface markers expressed by MPs are crucial for their detection in flow cytometry according to their cellular origin and support the use of patterns of expression of specific subpopulations of MPs as markers for the onset and progression of disease.

MPs can also carry membrane receptors and ligands, in addition to cytosolic proteins such as transcription factors and enzymes that are functionally bioactive, including for instance phosphatidylinositol-3-kinase (PI-3K) and mitogen-activated protein kinase (MAPK) [2]. The identification of specific and differentially expressed surface markers between MPs derived from healthy and abnormal cells, such as dysfunctional vascular cells, would provide tools for better disease diagnosis and progression monitoring.

### 3.2. Lipid Content of MPs

MPs consist of a lipidic bilayer membrane identical to the parent cell plasma membrane. Membranes of MPs compared to cell plasma membranes have higher flip-flop motions which facilitate the distribution and exchange of phospholipids between the membrane leaflets [2, 39]. MP membranes are shown to exhibit a high content of sphingomyelin and cholesterol which eventually contributes to their rigidity and resistance to degradation making them thus ideal carriers for various macromolecules including proteins and nucleic acids such as miRNAs [40, 41]. Macrovesicles can harbor also enzymes implicated in the metabolism of lipids such as phospholipases A2, C, and D and carry a variety of bioactive lipids such as free fatty acids which can modulate cellular responses in recipient cells [42]. The differential lipid composition of MPs may reflect the conditions of stimulation of the parent cell and vary according to the content in lipids in the extracellular environment surrounding the emitting cells.

It is anticipated that MPs from subjects suffering from metabolic disturbances and dyslipidemias may have a specific lipid and lipid metabolic enzyme composition compared to MPs from healthy subjects, which may eventually contribute to a differential effect of these MPs on recipient cells; however, this hypothesis warrants further investigation. The numbers of MPs from platelets [43] and those from T cells or neutrophils and those harboring TF [44] were found to have a negative correlation with plasma levels of plasma HDL cholesterol.

### 3.3. Nucleic Acids and MicroRNA Content of MPs

In addition to carrying bioactive lipids, membrane, and cytoplasmic proteins and other organelles and nuclear components from the parent cell, MPs can also carry nucleic acids, specifically mRNA and microRNA (miRNA), suggesting MPs can deliver genetic material to recipient cells [5]. MicroRNAs, which are small noncoding RNAs, play a key role in the epigenetic regulation of gene expression and control many metabolic and physiological processes associated with health and disease. Circulating MPs appear to be an efficient carrier for miRNA and a tool for their transfer to target cells where they can modify the physiological response of the cell.

It has been reported that platelets carry a rich and diverse content of more than 400 miRNAs [45, 46]. Importantly, the miRNA cargo content of platelet-derived MPs differs under disease conditions and upon cellular activation [47, 48]. The treatment of cells with thrombin, a potent platelet agonist, altered the miRNA signature of platelets and platelet-derived MPs with differential expression of miR-15a, miR-339-3p, miR-365, miR-495, miR-98, and miR-361-3p being reported [46, 47]. It is now becoming apparent that diabetes induces its own miRNA signature which may contribute to the onset of diabetes-induced disturbances. The pattern of expression of 5 plasma miRNAs, miR-15a, miR28-3p, miR-126, miR-223, and miR-320, was found to form a unique molecular signature enough to differentiate between individuals with and without diabetes [49]. Laffont et al. [47] reported that when platelets were activated with thrombin release, they selectively and preferentially released MPs rich in miR-223 [47]. Recently, Jansen et al. [50] reported in a cohort composed of 80 control subjects and 55 patients suffering from type-2 diabetes that endothelial-derived MPs from diabetics had a lower expression of miR-126 and miR-26a compared to controls. Intracellular miR-126 expression level was reported to define the regenerative capacity and proangiogenic capacity of CD34^+^ peripheral blood mononuclear cells [51] and circulating angiogenic early outgrowth cells [52].

## 4. Methods of Detection of MPs

Using a variety of methods, it is quite easy to detect MPs in body fluids. These techniques include assessing the concentration of proteins carried by MPs, flow cytometry analysis using cell-specific antigen markers, or ELISA assays to detect the prothrombotic activity of MPs based on the assumption that all MPs expose PS at their surface and can thus interact with annexin V. More recently, new single-particle detection instruments have been used for the analysis of MPs, including the resistive pulse sensing (RPS) and the nanoparticle tracking analysis (NTA), which can measure the size distribution and the concentration of in-solution MPs [53, 54]. Because of the tendency of MPs, especially those of a platelet origin, to aggregate during the process of isolation by centrifugation, single-particle detection instruments (e.g., RPS and NTA) can detect a lower concentration of MPs. However, flow cytometers which are particle size-insensitive instruments can detect these large aggregates, and thus, a higher concentration is detected [53, 54].

Flow cytometry analysis is the most preferred technology nowadays to trace MPs because of its high speed of detection and the availability of more sensitive machines. With flow cytometry, there is a possibility to determine the total number of MPs as well as the cellular origins of MPs by targeting specific surface antigens expressed by MP particles from a specific cell origin. However, owing to the differences between flow cytometers in terms of their optical configuration and sensitivity of detection and because of the absence of standard units for the measurement of MPs, the comparison of data between laboratories is difficult which hinders the potential use of MPs as a diagnostic and/or prognostic tool for diseases due to the variability and nonreproducibility of results depending on the machine used [53].

The need for standardization of techniques of detection of MPs by flow cytometry was recognized by the Scientific Standardization Committee on Vascular Biology (SSCVB) of the International Society on Thrombosis and Hemostasis (ISTH), which launched an initiative over 6 years ago to standardize MP measurements using flow cytometry following a survey that highlighted that nearly 75% of laboratories use flow cytometry to count and phenotype MPs according to cellular origins in clinical samples (e.g., plasma). An inaugural collaborative workshop was established to determine both the resolution and the level of background noise of major flow cytometers used across several research laboratories. The other objective of this workshop was to define the reproducibility of platelet-derived MP enumeration in human plasma between different instruments. The strategy followed was to use Megamix® fluorescent calibrated beads to allow for a reproducible setup of MP window analysis [55].

Lacroix et al. [56] tested 49 flow cytometers in 40 laboratories and found that the instruments tested had very heterogeneous forward scatter (FS)/FS channeling (FSC) resolution and machine background noise. The authors also reported that the use of Megamix® helped in identifying and fixing some of the parameters affecting FS/FSC resolution and that eventually 33 instruments tested were validated [56]. Nonetheless, because of the huge variety of optical designs in flow cytometers available on the market, it was possible to achieve an efficient universal standardization methodology to count platelet-derived MPs. The consortium observed, however, that the resolution was better and more homogenous in a subset of instruments using side scatter (SSC) instead of FSC. Thus, another set of beads was chosen to better respect the design of SSC-focused flow cytometers [57].

More recently, another workshop was organized by the SSCVB to test a new strategy for the standardization of MP detection by flow cytometers and to evaluate interinstrument reproducibility in detecting platelet-derived MPs. This new strategy used two different types of beads to suit instruments of different optical designs (Megamix-Plus® FSC or SSC beads). The study by Cointe et al. [58] included 44 laboratories across 17 countries and had 52 instruments registered for testing. Flow cytometers qualified for inclusion in the strategy of scandalization according to their resolution and low levels of background noise. All these instruments could correctly rank levels of platelet-derived MPs in a plasma. The interlaboratory variance in the enumeration of MPs was between 28 and 37%. The authors showed that the use of size-calibrated beads can successfully be utilized to standardize MP counting across different instruments and laboratories if special care is taken to consider the internal instrument behavior for size-related measurements and this independently of using FSC or SSC as the relative sizing parameter [58]. Despite this second study which only investigated parameters for the detection of platelet-derived MPs and only focused on the optimal scatter-based gating of the MP population, it is a very important initiative and an important step forward standardizing conditions of detection of MPs by flow cytometry.

One major challenge in using flow cytometers in detecting MPs is still the limit of the detection, and further technological developments are thus required to improve the detection of smaller MPs. Furthermore, additional efforts from the extracellular vesicle community are still warranted to improve standardization methods for MP detection for an optimal use of circulating MPs as routine laboratory diagnosis and/or prognosis tools.

## 5. MPs as Biomarkers of Cardiovascular Diseases

Increased numbers of plasma MPs were reported in several cardiovascular diseases associated with insulin resistance, inflammatory, and/or procoagulant states [5]. In these pathologies, platelet-derived MPs were found to represent the dominant subpopulation. However, MPs from other origins including endothelial cells, erythrocytes, or leukocytes were also found to be increased in certain pathological states such as metabolic syndrome [16], acute coronary syndrome [59, 60], severe hypertension [61], and type-2 diabetes [62]. Two key features of these disease states are endothelial dysfunction and impaired microcirculation with which the levels of circulating MPs frequently correlate indicating that MPs carry cellular messages that may contribute to the onset and progression of these pathologies.

Circulating MPs found in the plasma originate from vessel wall cells such as endothelial and smooth muscle cells and other circulating cells such as platelets, erythrocytes, and leukocytes. However, most of these MPs are derived from platelets which represent nearly 80% of the total population of circulating MPs. An important physiological role of platelets is the control of hemostasis by providing a membrane surface to enhance blood coagulation and promote the generation of the fibrin network in the hemostatic plug [63]. Platelets have a very high rate of scrambling of membrane lipids and when stimulated generate MPs most rapidly especially those exposing PS which contributes in preventing bleeding; however, an excessive shedding of MPs may cause thrombotic complications [12]. Platelets produce MPs which display a heightened prothrombin-converting activity and which provide an extended membrane surface to promote coagulation. It has been reported that MPs originating from platelets are 50- to 100-fold more procoagulant than activated platelets [13]. However, although high circulating levels of platelet-derived MPs were reported in many disease conditions, changes in nonplatelet-derived MPs, particularly those originating from endothelial cells, were found to contribute a key role in the pathogenesis of these disorders [15–18].

MPs are recognized today as promising biomarkers for the diagnosis and monitoring of disease progression because total levels of MPs or those from specific cellular origins were found to correlate with the severity or the progression of several disorders. An increase in the numbers of MPs from a specific cell population may represent a signature of injury and a marker of changes to which these cells are subjected to. For instance, endothelial MP levels were found to be good predictors for the health of the endothelium and to correlate with endothelial dysfunction. For example, high levels of endothelial MPs were found to correlate positively with arterial erectile dysfunction in patients with insulin resistance [64] and with endothelial dysfunction in patients suffering from chronic ischemic left ventricular dysfunction [65]. The circulating levels of platelet-released MPs were associated with carotid and intima/media thickness and corporal in mass in obese subjects [66]. Activated leukocyte-derived MPs (CD62L^+^) were found to positively correlate with oxyhemoglobin desaturation index (ODI) in patients with obstructive sleep apnea which do not suffer from any cardiovascular comorbidities [18].

## 6. Circulating MPs in Insulin-Resistant States and Diabetes

### 6.1. Impact of Diabetes on MP Expression Profile

Much evidence shows that circulating numbers of MPs are more elevated in patients with diabetes and in experimental animal models of diabetes and they contribute to the development of cardiovascular complications associated with diabetes. A meta-analysis study by Li et al. [67] investigated the relationship of MPs with type-2 diabetes. The authors included 48 studies with a total number of 2,460 patients suffering from type-2 diabetes and 1,880 nondiseased volunteers, of which 34 studies were quantitatively assessed. The general analysis revealed that type-2 diabetes patients, regardless whether they had comorbidities or not, exhibited higher circulating levels of total MPs, platelet-, monocyte-, and endothelium-derived MPs in comparison to healthy volunteers. However, the count of leukocyte-derived MPs was similar between controls and patients [67].

Previously, Sabatier et al. [68] investigated circulating levels of MPs and determined their procoagulant properties in both type-1 and 2 diabetics and reported in general all diabetics had higher numbers of MPs compared to controls. However, the authors observed that diabetic patients exhibited a difference in the pattern of expression of MPs according to their cell origins and in their prothrombotic action. Patients with type-1 diabetes had higher levels of MPs derived from platelets and endothelial cells in addition to prothrombotic MPs compared to controls and type-2 diabetes patients [68]. Later, Feng et al. [69] reported that type-2 diabetes patients had elevated levels of procoagulant, platelet, leukocyte, and endothelial MPs compared to controls and endothelial MPs were the only population to positively correlate with endothelial dysfunction as assessed by flow-mediated dilation (FMD) in patients [69].

Koga et al. [70] stressed the importance of endothelial MPs as markers of vascular dysfunction in diabetes and showed that endothelial MPs were twofold higher in diabetics compared to control volunteers and that these MPs have even higher levels of diabetic patients suffering from coronary heart disease than those free from such a comorbidity [70]. In another study, Bernard et al. [71] evaluated the association between plasma levels of endothelial- and platelet-derived MPs and the presence of coronary noncalcified plaques in patients with diabetes. Researchers reported that MPs of endothelial origin (CD144^+^) were highly elevated in patients exhibiting coronary noncalcified plaque [71]. Tsimerman et al. [72] reported that diabetic patients had high circulating levels of MPs originating from platelets and that patients also suffering from diabetic foot ulcers had the highest numbers of prothrombotic, endothelial, and platelet MPs compared to patients suffering from diabetes and coronary heart disease or retinopathy [72].

A study by Zhang and coworkers [73] investigated the impact of the copresence or not of obesity and type-2 diabetes on the profile of expression of circulating MPs. The authors reported that regardless of the presence or not of obesity, type-2 diabetics had high levels of total platelet-derived MPs or those from activated platelets which express fibrinogen, tissue factor (TF), or P-selectin. However, investigators did not find any specific effect of obesity on MP counts in the absence of type-2 diabetes [73]. The absence of differences in the presence of obesity alone is contradicting with previous reports indicating an effect of obesity on the numbers of circulating MPs [5]. This is potentially due to the small cohort of patients included in this study (5 to 11 patients per group) [73].

### 6.2. MP Expression in Type-2 Diabetes and Asymptomatic Atherosclerosis

Recently, Berezin et al. [74] investigated the phenotype of expression of circulating MPs between healthy volunteers and patients with type-2 diabetes either with angiographic evidence of asymptomatic atherosclerosis or without any known coronary atherosclerosis. The authors found that in comparison to controls and diabetics with no angiographic evidence of atherosclerosis, patients with asymptomatic atherosclerosis had elevated numbers of circulating platelet-derived (CD41a^+^) and endothelial-derived (CD144^+^/CD31^+^ or CD144^+^) MPs in addition to MPs from procoagulant endothelial cells (CD31^+^/annexin V^+^). Using a multivariate regression model, the authors found that MPs from procoagulant endothelial cells (CD31^+^/annexin V^+^) were an independent predictor for nonsymptomatic atherosclerosis [74].

### 6.3. Differential MP Origins and Endothelial Dysfunction in Obesity

High circulating levels of MPs from different origins were also observed in various studies of patients with obesity, which is closely associated with insulin resistance and considered number one contributor to the development of type-2 diabetes. Several studies in obese children suggested a predictive potential for MPs for future adverse cardiometabolic events. A cross-sectional study by Bruyndonckx et al. [75] investigated the relationship between microvascular endothelial dysfunction and numbers of MPs derived from endothelial cells and endothelial progenitor cells (EPCs) in a cohort composed of 57 obese and 30 age- and sex-matched and normal weight children. The noninvasive measurement of microvascular endothelial function by determining the peripheral arterial tonometry at the distal phalanx, that reflects endothelial function at the level of small resistance arteries, revealed that obese children had significantly impaired peripheral endothelial function in addition to a lower number of circulating EPCs and a high level of circulating endothelial-derived MPs (CD31^+^/CD42b^−^) compared to control children. Single regression and multivariate analysis showed that the peak response (or the ratio between postocclusion pulse-wave amplitude over the baseline amplitude) correlated negatively with endothelial-derived MPs but positively with EPCs count that were both independent determinants of the peak response together with systolic blood pressure [75].

### 6.4. MP Expression Profile in Metabolic Syndrome

Obesity is often associated with other comorbidities including insulin resistance, type-2 diabetes, dyslipidemias, and hypertension. This association of cardiometabolic disturbances is commonly referred to as the metabolic syndrome (MetS) [76, 77]. The pathophysiology of MetS is tightly related to insulin resistance and excess of fatty acids [78, 79] in addition to low-grade inflammation [77, 79, 80]. The combination of inflammatory factors with excessive production of reactive oxygen species (ROS) contributes to the development of cardiovascular disturbances in MetS [5]. Several studies investigated the count and phenotype of circulating MPs present in the plasma from patients with MetS. Agouni et al. [16] determined the count and phenotyped plasma MPs according to their cellular origins in 43 MetS patients and 37 volunteers. It was observed that MetS patients had higher numbers of total MPs in comparison to controls, in addition to prothrombotic MPs (annexin V^+^) and those originating from platelets, endothelial cells, and erythrocytes [16]. These observations were also corroborated by the work of Helal et al. [81] in a bicentric cohort study of MetS patients. The authors also found that BMI correlated positively with the number of prothrombotic and endothelial-derived MPs [81].

These studies strongly indicate that MPs are potential biomarkers for the diagnosis, stratification, and follow-up of disease progression and development of cardiovascular comorbidities for metabolic disorders where insulin resistance is a central pathophysiological driver such as obesity, diabetes, and MetS. These findings also highlight the crucial role that MPs may play in the development of metabolic diseases and in the onset and/or maintenance of cardiovascular complications associated with these pathological states.

## 7. Pathophysiological Roles of MPs in Altering Cell Signaling and Causing Cell Injury in Insulin-Resistant States and Diabetes

In the last decade, many studies were conducted to investigate the role of MPs as active effectors in the onset and progression of diabetic cardiovascular complications. Cells subjected to pathological stimuli have modified intracellular responses, and thus, MPs generated from these cells can transfer these deleterious biological messages to recipient cells to modify in turn their cellular responses.

**Figure 1:**
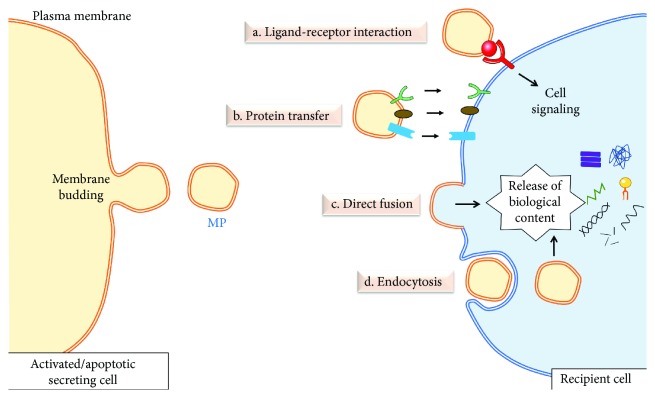
Mechanisms of interaction of MPs with recipient cells. MPs are first released from activated or apoptotic cells by direct budding of the plasma membrane. MPs can then directly interact with the target cell via ligand-receptor interaction which results in cell signaling (a), transfer proteins (i.e., adhesion molecules, MHC, and membrane receptors) from the MP vesicle to the surface of the target cell (b), or deliver the biological content of the MP to the target cell through either direct fusion of the MP with the plasma membrane of the target cell (c) or endocytosis (d). PS: phosphatidylserine, MHC: major histocompatibility complex, MP: microparticle.

MPs can transfer biological information to recipient cells through multiple potential mechanisms although the exact process is not fully understood yet. These mechanisms of interaction with target cells may involve the following: (i) direct interaction between surface proteins in a ligand/receptor type of interaction; (ii) transfer to target cell of surface receptors, ligands, channels, proteins, genetic material, and lipids; (iii) merging between membranes of MPs and target cells; and (iv) internalization of MPs inside the target cell. The mechanism of interaction with recipient cell involving ligand and receptor interaction may rapidly either switch on or switch off intracellular signaling responses; however, the other means of interaction will involve more complex changes of cellular responses inside target cells. MPs can also interact with recipient cells through more than one of these mechanisms. For instance, it has been previously reported that MPs carrying the morphogen Shh were able to activate the morphogen's signaling pathway to enhance endothelial angiogenic process and NO release [7, 82]. However, two hours following this surface interaction, MPs carrying Shh were internalized by endothelial cells and enhanced the expression of antioxidant enzymes [83].  Figure 1 summarizes the mechanisms of interaction of MPs with recipient cells.

In the next section, we will discuss the role of MPs in the onset and maintenance of cellular dysfunction and injury associated with insulin resistant states and diabetes with special emphasis on the molecular mechanisms involved. The major alterations in circulating levels of MPs and their molecular contribution to diabetes-induced complications in humans and *in vivo* and *ex vivo* models are summerized in Table 2.

### 7.1. MPs and Vascular Dysfunction

Due to its direct exposure to blood stream, one major target of circulating MPs are endothelial cells, which play an important role in the maintenance of vascular homeostasis by achieving a balance between vasoactive agents (vasodilators and vasoconstrictors) and prothrombotic and antithrombotic factors in addition to a tight control of vascular permeability among other key actions. Physiological levels of shear stress regulate the homeostasis of endothelial cells; however, extreme disturbances in shear stress due to changes in vascular pressure can imbalance the release of endothelium-derived factors leading to endothelial dysfunction.

Endothelial dysfunction is referred to as the reduced capacity of vessels to dilate in response to the activation of endothelial cells by humoral or mechanical mediators such as shear stress or bradykinin. It is now widely recognized that endothelial dysfunction is an independent predictor of future adverse cardiovascular events which are widely associated with obesity and diabetes. Most notably, a decrease of endothelial NO production and bioavailability, a heightened release of vasoconstrictor mediators, and an enhanced oxidative stress, which altogether lead to alterations of vascular reactivity, cause vascular inflammation and vascular remodeling by affecting the levels of proteins and enzymes involved in these processes. It is expected that MPs may affect all these vascular events that contribute to endothelial dysfunction.

MPs produced from activated vascular cells, such as endothelial cells, are biomarkers of cell injury; however, they are also capable of interacting with neighboring cells in an autocrine or paracrine mechanism and modify therefore the cellular responses of target cells including the emitting cells themselves. Jenkins et al. [84] reported that disturbed blood flow in the forearm of healthy men by distal cuff occlusion was shown to increase endothelial MPs in the experimental arm by nearly 9-fold after 20 minutes only, while activated endothelial MPs expressing E-selectin (CD62E^+^) increased by 4-fold [84]. Even though arteries in the forearm are less prone to atherosclerosis, it was possible to observe acute changes in activated endothelial MPs, suggesting that in atherosusceptible arteries from the elderly or individuals suffering from comorbidities that are associated with impaired endogenous endothelial repair mechanisms (e.g., obesity or type-2 diabetes), the effects might be more severe [84]. The acute reduction of physical activity for 5 consecutive days was also reported to increase the numbers of total and activated endothelial-derived MPs in healthy volunteers, which was associated with an impairment in popliteal artery flow-mediated dilation (FMD) [85]. These studies indicate that acute changes in MP release not only are early biomarkers of cell injury but can also be considered active players and conveyers of biological message which contribute to changes in the vascular function.

Several studies have reported a relationship between numbers of total MPs or specific cell populations and endothelial dysfunction; however, few studies looked at the molecular mechanisms underpinning the actions of MPs on endothelial cells. Ishida et al. [86] found that streptozotocin-injected rats (a model of type-1 diabetes) had high numbers of plasma MPs originating from platelets (CD61^+^) and activated platelets (CD62P^+^) in addition to procoagulant MPs (annexin V^+^) compared to control animals. They also found that the *ex vivo* exposure of carotids from control rats to diabetic MPs reduced endothelium-dependent relaxation by blunting eNOS protein expression [86]. Using a diet-induced obesity rat model, Heinrich et al. [87] reported that obese rats had higher circulating MP levels compared to chow diet-fed animals, including those of procoagulant, endothelial, platelet, leukocyte, monocyte, and T lymphocyte origins. The incubation of endothelial cells *in vitro* with MPs from obese rats enhanced the expression of vascular cell adhesion molecule 1 (VCAM-1) and increased oxidative stress indicating that MPs from insulin resistant animals induced endothelial dysfunction [87].

Previously, Martin et al. [88] observed that MPs engineered *in vitro* from apoptotic T lymphocytes reduced endothelium-dependent relaxation and impaired shear-mediated dilation in mouse aortas and small mesenteric arteries, respectively. These effects were related to a deceased expression of eNOS and overexpression of caveloin-1, a negative regulator of eNOS. Furthermore, the authors found that MPs derived from diabetic T lymphocytes or circulating total MPs from diabetic patients blunted eNOS protein expression in HUVECs [88]. Tesse and coworkers [89] have shown later that *ex vivo* exposure of mouse aortic rings to MPs from either apoptotic T lymphocytes or patients with diabetes caused impaired vascular response to vasoconstrictor agents. This effect was linked to an enhanced release of NO and prostacyclin mediated by NF-*κ*B-induced upregulation of inducible forms of NOS (iNOS) and cyclooxygenase (COX-2). The authors also observed that *in vivo* injection of mice with MPs derived from apoptotic T lymphocytes impaired vascular response to vasoconstrictors, which was prevented by the concomitant administration of both NO and COX-2 inhibitors [89]. Interestingly, Tesse et al. [89] also observed that the vascular effects mediated by diabetic and T lymphocyte-derived MPs involved the interaction of Fas-ligand (FasL or CD95L) carried out by MPs its receptor (Fas or CD95) expressed by smooth muscle cells [89].

Agouni et al. [16] observed that MPs collected from patients suffering from MetS reduced endothelium-mediated vasodilation in aortas of mice that received an intravenous injection of MetS MPs in comparison to those that received MPs from MetS-free subjects. Of interest, the authors found that in spite the majority of MPs from MetS patients originated from platelets, the effects mediated by MetS MPs on endothelial function were mainly caused by MPs of nonplatelet origin [16]. The same group has subsequently found that MetS MPs could also interact with smooth muscle cells to cause vascular dysfunction [15]. The team found that MetS MPs injected into healthy mice impaired vasocontractile response in the aorta by stimulating an inflammatory response in the vessel through the overexpression of iNOS increasing thus vascular NO production [15].

More recently, Safiedeen et al. [90] have reported that MPs engineered *in vitro* from apoptotic T lymphocytes or obtained from MetS patients activated the proinflammatory signaling pathway of endoplasmic reticulum (ER) stress, in cultured human aortic endothelial cells (HAECs) and *in vivo* in mouse aortas, which contributed to MP-induced endothelial dysfunction [90]. Protein synthesis and key posttranslational modifications occur inside the ER. In situations associated with an increased demand on protein synthesis such as obesity or diabetes, there is an overload on the ER leading to the accumulation of misfolded and/or unfolded proteins within the ER lumen that results in the activation of the unfolded protein response (UPR) with the primary goal to improve ER homeostasis and clear the protein load that has accumulated to improve cell viability and survival [91, 92]. However, when the activation of the UPR pathway prolongs and becomes chronically switched on, this can lead to a situation of “ER stress,” which is associated with the activation of adverse cellular responses that may lead to cell death [91, 92]. Several reports linked ER stress response activation to the onset of insulin resistance in obesity and diabetes [93, 94]. ER stress is also tightly associated with the activation of major inflammatory intracellular signaling response such as nuclear factor (NF)-*κ*B and c-Jun N-terminal kinase (JNK). The extreme activation of ER stress may cause apoptosis [91, 92, 95, 96].

Endothelial cells undergoing apoptosis are dysfunctional, more procoagulant, and prone to adhere to platelets [97]. ER stress-mediated apoptosis involves, at least, three apoptotic subpathways: the first is through the activation of proapoptotic transcription factor C/EBP homologous protein (CHOP), the second is controlled by the activation of JNK signaling response, and the third is due to the activation of ER-associated caspases 3 and 12 (only found in rodents) [92, 95, 96]. ER stress response was shown to be activated in endothelial cells lining vascular areas with the highest susceptibility to develop atherosclerotic plaques and hence indicate an involvement of this pathway in the development of endothelial dysfunction [98].

Recently, it has been shown that high glucose disturbed NO signaling and impaired angiogenic capacity of cultured HUVECs through the activation of ER stress response [99]. Interestingly, Safiedeen et al. [90] reported that the exposure of HAECs to MetS or apoptotic T lymphocyte-derived MPs caused the activation of serval key ER stress markers, including the nuclear translocation of activating transcription factor (ATF)-6, and reduced endothelial NO production [90]. All these effects were interestingly prevented by the pretreatment of cells with a chemical chaperone, tauroursodeoxycholic acid (TUDCA), to improve the native functions of the ER and improve protein folding [90]. The intravenous injection of MetS or apoptotic T lymphocyte-derived MPs into mice impaired endothelium-mediated vasodilation in the aorta; however, the preinjection of animals with TUDCA prevented this deleterious effect on vascular function. The authors also found that the effects of MetS and apoptotic T lymphocyte-derived MPs on the activation of ER stress in HAECs involved two membrane receptor interactions, Fas/FasL and low-density lipoprotein receptor (LDL-R), that were both been previously found to be implicated in the vascular effects of MetS or apoptotic T lymphocytes-derived MPs [15, 89, 100]. Furthermore, MetS and apoptotic T lymphocyte-derived MPs increased the protein expression of the neutral sphingomyelinase (SMase) that was found to contribute to the activation of ER stress response [101]. The neutralization of FasL expressed at the surface of MPs or the blockade of the LDL-R expressed at the surface of endothelial cells normalized the levels of protein of expression of SMase. Furthermore, the blockade of SMase action reversed the actions of MPs on NO signaling and ER stress response activation, suggesting that the effects of MPs are mediated through neutral SMase [90].

Altogether, these studies highlight the complex interactions of MPs from insulin resistant or diabetic patients with cells of the vessel wall to induce vascular dysfunction which eventually contributes to the development of comorbidities associated with these metabolic disorders. The deeper understanding of the molecular mechanisms involved will pave the way for the identification of novel therapeutic targets to reduce the burden of these cardiometabolic disturbances on the quality of patients' lives.

### 7.2. MPs and Ischemic Diseases

MPs are potential vectors of biological messages between cells and were found to play key roles in regulating angiogenesis in ischemic conditions. Numerous studies have demonstrated that human T lymphocyte-derived MPs carrying the morphogen Shh were able to regulate the proangiogenic activity of endothelial cells *in vitro* through the regulation of the expression of proangiogenic genes [82, 83]. Importantly, the proangiogenic effects of these MPs were further confirmed *in vivo* using mouse models of hindlimb ischemia. The authors found that the treatment of the ischemic mouse with MPs bearing Shh enhanced the postischemic reparative neovascularization by regulating NO pathway and modulating angiogenesis-related gene expression in these models [102]. In another study using an *in vivo* mouse model of angiogenesis, Benameur et al. [103] observed that MPs carrying peroxisome proliferator-activated receptor (PPAR)-*α* play a stimulatory role on neovascularization mediated by EPCs originating from the bone marrow. The MP-induced angiogenic effects were essentially mediated by the improvement of EPC differentiation and the enhancement of the proangiogenic activity of endothelial cells [103].

Diabetes is associated with a high risk of ischemic diseases and other associated complications as discussed in this review. Given the increasing clinical significance of MPs in various pathologies and their important biological functions as well as the importance of other cell-derived molecular partners, a growing number of studies have started investigating the contribution of MP-derived miRNA transfer in modulating the pathogenesis of many diseases. MPs harboring miRNA have been reported in multiple diseases including cancer and cardiovascular disorders [104]. It was observed that the expression of endothelial-specific miRNA molecules in MPs shed from endothelial cells was significantly altered in diabetes mellitus with stable coronary artery diseases. In addition, endothelial-derived MPs bearing miR-126 were shown to promote vascular endothelial repair through the transfer of miRNA to the target cells. This effect was abolished under hyperglycemic conditions [105]. In another translational approach, it was further confirmed that diabetes mellitus correlated with impaired expression of not only endothelial miR-126 but also miR-26 in circulating MPs when compared to patients without diabetes mellitus. These results underscore the potential implication of endothelial-derived MPs in modulating vascular biology and consequently leading to diabetes mellitus vasculopathy [50]. Altogether, these findings support that diabetics with lower levels of miR-126 and miR-26 can be at a higher risk of concomitant coronary artery disease.

Consistent with previous findings published by Caporali et al. [106], a more recent research has provided a new level of understanding of the contribution of MPs to the development of diabetes-mediated vascular complications, with particular emphasis on the interaction between endothelial cells and pericytes in a rodent model of hindlimb ischemia. Results demonstrated that MPs secreted from diabetic endothelial cells and carrying miR-503 were subsequently delivered to neighboring pericytes to modify the permeability of vessels and alter the postischemic angiogenic process in limb muscles via the regulation of genes under the control of miR-503, such as VEGFA and EFNB2 (Ephrin B2) [107].

It has also been observed that *db/db* mice, a genetic rodent model of type-2 diabetes, exhibited elevated plasma MPs originating from endothelial cells compared to wild-type mice and that these MPs caused a reduction in cerebral microvascular density [108]. The authors found that MPs collected from *db/db* animals also impaired the properties of EPCs as evidenced by the failure of EPCs (obtained from control mice) prestimulated with MPs collected from *db/db* to improve ischemic damage following their administration into diabetic mice [108].

Because of the importance of tissue factor (TF) in regulating angiogenesis, the crucial role of MPs derived from microvascular endothelium that bear TF was demonstrated in enhancing collateral flow and capillary formation in a murine hindlimb ischemia model [109, 110]. TF-bearing microvascular endothelial-derived MPs accelerated the angiogenic process through a paracrine control of adjacent endothelial cells, via a mechanism implicating the *β*1-integrin pathway, Rac1-ERK1/2-ETS1 to form new and functional mature vessels. Furthermore, Edrissi et al. [111] reported that MPs shed during cerebral ischemia enhanced the permeability of endothelial cell layer. This increase in endothelial permeability was, at least, mediated by an accelerated apoptosis induced by the transfer of the activated TNF*α* pathway molecules, caspase 3, and Rho kinase delivered by MPs to the target cells [111]. Tsimerman et al. [72] also observed that MPs obtained from patients suffering from diabetes enhanced the procoagulant activity of endothelial cells. The prothrombotic activity was the highest in endothelial cells stimulated by MPs collected from diabetic patients with severe diabetic foot ulcers, suggesting a role for these MPs in thrombotic complications associated with diabetes [72].

These findings open up intriguing new therapeutic strategies based on the use of MPs as potential novel therapeutic tools in the treatment of critical limb ischemia and other ischemic diseases. Taking into account the different properties of MPs, much evidence supports the concept of expanding the MPs' use as efficient therapeutic tools in altered angiogenesis associated with ischemic diseases [112].

### 7.3. MPs and Myocardial Infarction

Diabetic patients are at a high risk of developing acute coronary syndromes (ACS). Due to their procoagulant and proinflammatory potential, MPs were found to contribute to the formation of thrombus and the progression of atherosclerotic disease. High circulating levels of MPs have been observed in ACS [113, 114]. ACS include unstable angina, non-ST segment elevation myocardial infarction (NSTEMI), and ST-segment elevation myocardial infarction (STEMI) in addition to sudden death. ACS are frequently characterized by the buildup of vulnerable atherosclerotic plaques in coronary arteries and manifest following the plaque rupture and the occlusion of arteries by the formed thrombi [115].

Several studies reported high circulating levels of MPs from various cell origins in STEMI patients and found that their levels reflected systemic inflammation and cell injury and activation in addition to correlating with the size of ischemic cardiac tissue [116–120]. For instance, Porto et al. [116] observed that STEMI patients had higher levels of platelet- and endothelial-derived MPs in intracoronary blood compared to aortic blood and that these MPs correlated positively with thrombus scores [116]. Furthermore, it has been reported that the numbers of circulating MPs continued to increase in the short term following appropriate intervention in STEMI patients. Zhou et al. [117] followed up circulating levels of MPs over time in STEMI patients undergoing percutaneous transluminal coronary intervention (PCI) (before and up to 48 h after PCI). The authors found that platelet MPs increased directly after the PCI and continued to rise over time until the end of follow-up period, whereas the levels of leukocyte- and endothelial-derived MPs decreased after the PCI, but rose thereafter until the end of the follow-up period [117].

Recently, Chiva-Blanch et al. [118] compared the profile of MP expression between STEMI and NSTEMI patients. The authors found that STEMI patients had higher numbers of platelet-derived MPs compared to NSTEMI in spite of receiving a dual antiplatelet treatment, indicating that the process of platelet activation may be more implicated in the pathogenesis of STEMI. STEMI patients exhibited a higher plasma TF procoagulant activity compared to NSTEMI patients, which positively correlated with the number of MPs originating from platelets, monocytes, and those harboring TF [118].

However, MPs are not always playing a deleterious role in cardiac ischemic disease. The process of remote ischemic conditioning (RIC) is a good example for this. RIC consists of subjecting a remote area of heart muscle to brief reversible cycles of ischemia and reperfusion, conferring a general protection to distant tissues and organs, which become resistant to injury caused by prolonged episodes of ischemia and reperfusion [121]. Despite the fact that effector pathways of RIC were described in the literature, the propagation patterns of cardioprotective signals between organs remain unclear [122]. Interestingly, it was shown that the release of MPs from the heart increased following a preconditioning procedure and these MPs were found to contribute to the forwarding of protective remote conditioning signals. This suggests that MPs exert a protective effect in ischemic heart diseases [123]. Very recently, Bueno-Betí et al. [33] reported that MPs engineered *in vitro* and harboring the morphogen Shh restored the vasculogenic properties of EPCs obtained from patients suffering from myocardial infarction to the levels of healthy volunteers [33].

### 7.4. MPs and Ischemic Stroke

Diabetes mellitus is recognized as an important risk factor for stroke and is associated with increased incidence of ischemic stroke at all ages [124, 125]; however, the chronic influence of stroke on cell activation and dysfunction processes remains poorly understood. Circulating levels of neural precursor cell (NCP)-derived MPs (CD56^+^/CD34^+^/annexin V^+^) were found to be higher in stroke patients in comparison to high cardiovascular risk controls. This increase was chronically maintained when compared to the levels measured at the onset of attacks in patients with lower lesion volumes. However, a decreased level of NCP-derived MPs and increased levels of smooth muscle cell-derived MPs were observed in patients with higher volume of cerebral lesions. These data suggest that NCP-derived MPs can reflect an ongoing repair of the damaged brain tissue in patients and can be considered as a potential biomarker of stroke.

The specific underlying pathophysiological mechanisms of the involvement of MPs in stroke need further investigations [126]. MPs harboring TF (TF^+^-MPs) are produced in various pathological conditions including atherosclerosis, cancer, acute coronary syndrome, and diabetes where they can trigger thrombosis cascade [127]. It is well documented that the presence of TF on MPs markedly increased their procoagulant activity [128]. In a study of acute ischemic stroke, 26% of stroke patients had diabetes; the authors reported significantly higher plasma numbers of TF^+^-MPs and an elevated circulating concentration of TF pathway inhibitor (TFPI) in stroke patients compared to healthy controls. Moreover, one week following diagnosis, the activity of MPs was more elevated in stroke patients not treated with a tissue plasminogen activator (Alteplase) compared to their activity at the stroke attacks onset [129].

### 7.5. MPs and Diabetic Cardiomyopathy

Understanding of the underlying pathogenic mechanisms of diabetic cardiomyopathy is necessary for the early detection and control of this major cardiovascular complication of diabetes [130]. At early stages, diabetic cardiomyopathy is often unrecognized due to the lack of pathognomonic features. Indeed, these early stages are most often nonsymptomatic and only molecular and cellular alterations are taking place. Moreover, the insignificant alteration in the myocardial structure and function make the subclinical detection of diabetic cardiomyopathy extremely challenging in clinical practice. In addition, the subclinical state of the disease becomes more critical after an episode of ischemia. This will reduce the possibility to restore the heart function to normal level.

Interestingly, previous investigations provided evidence that miRNAs were found in human biofluids and considered as a novel potential category of biomarkers for type-2 diabetes [131] and suggested utilization of miRNA as promising biomarkers for the early diagnosis of subclinical diabetic cardiomyopathy [132, 133]. Promising preclinical studies suggested that the concept of miRNA and their specific antagonists could contribute to a better therapeutic control of heart failure to dyslipidemia, for example, in spite of the specificity and targeting challenges [134]. The potential of MP-contained miRNAs as a prominent biomarker for type-2 diabetes can be implicitly understood from the key roles that miRNAs play in the control of metabolic and cardiovascular pathophysiological processes [135].

The early detection of subclinical cardiomyopathy in diabetic patients using extracellular vesicles as biomarkers could be a future alternative of antidiabetic treatment. This can also serve as an early predictor of the diabetic myocardiopathy in patients with a high risk of diabetes-associated complications. Altogether, these new research streams highlight the crucial and emerging role of extracellular vesicles and their cargo in understanding cardiovascular physiology and the pathogenesis of cardiovascular disease associated with diabetes.

### 7.6. MPs and Diabetic Retinopathy

Diabetic retinopathy is another major, but common, complication of diabetes, which affects the microvasculature. It is an important contributor to vision loss and blindness in diabetic patients. Small vessels in the eye have pericytes that control the capillary tone, generate new capillaries, and protect the vessels against noxious molecules. Uncontrolled diabetes leads to the loss of pericytes and degradation of the blood retinal barrier which results in increased permeability and proliferation of endothelial cells [136].

Because of the association of diabetic retinopathy with progressive retinal capillary activation and proliferation, elevated vitreous levels of MPs from different cellular lineages were detected in patients with proliferative diabetic retinopathy. These MPs were especially of platelet, endothelial, and retinal origins. These MPs were able to stimulate cell migration and initiation of vessel formation, the key features of angiogeneis, in an *in vivo* Matrigel plug assay [137]. The levels of endothelial- and platelet-derived MPs were dramatically decreased in the vitreous body following a panretinal laser photocoagulation and after intravitreal treatment directed against vascular endothelial growth factor (VEGF), respectively [137]. These findings demonstrate to what extent MPs participate in the progression of proliferative diabetic retinopathy and highlight the role of MPs as indicators of therapy effectiveness and as efficient specific biomarkers for early diagnosis of retinal disturbances.

Different MP populations can exert different effects on the pathological neovascularization seen in diabetic retinopathy. For example, Tahiri et al. [138] have shown that lymphocyte-derived MPs (LMPs) suppressed laser-induced choroidal neovascularization (CNV). Given the fact that macrophages carry proangiogenic properties in CNV, LMPs were able to modulate the angiogenic properties of macrophages, suggesting that LMPs can be considered potent therapeutic antiangiogenic factors in pathological choroidal neovascularization [138]. Furthermore, extracellular vesicles released from mesenchymal stem cells that were treated with high glucose increased angiogenesis and retinal neovascularization *in vitro* by promoting the dissociation of pericytes from adjacent endothelial cells which results in uncontrolled proliferation of endothelial cells, contributing thus to proliferative diabetic retinopathy [139].

### 7.7. MPs and Diabetic Nephropathy

Diabetic nephropathy is characterized by the loss of renal podocytes. Podocytes are crucial for maintaining the proper permeability of the glomerular filtration barrier. Upon podocyte injury and loss, the glomerular filtration barrier's permeability is increased which results in the permeation of proteins such as albumin to the urine, a clinical manifestation of diabetic nephropathy. Since the loss of podocytes is irreversible, there is a need for a marker that can detect early cellular injury to podocytes which will allow for early intervention before clinical manifestation of symptoms. Burger et al. [140] indicated that podocytes can shed MPs into urine and that these MPs are significantly elevated in diabetic nephropathy in *in vitro* and *in vivo* models, even before the presence of albuminuria [140]. Similarly, Lytvyn et al. [141] found that patients with uncomplicated type-1 diabetes had significantly elevated numbers of podocyte-derived MPs in comparison to healthy volunteers [141]. It is clear that shedding of MPs from podocytes happens before the onset of diabetic nephropathy which supports their use as a diagnostic marker of renal injury.

Diabetic nephropathy is also an important risk factor for cardiovascular complications in diabetic patients. Endothelial dysfunction is believed to develop at the early stages of diabetic nephropathy and cardiovascular disorders. Accumulating evidence is demonstrating that certain MP populations that are significantly correlated with impaired vascular function are elevated in chromic kidney disease (CKD) and end-stage renal failure patients such as MPs from platelets and endothelial cells [142–144]. Endothelial MPs (CD144^+^ and CD31^+^/CD41^−^) strongly correlated with impaired vascular function *in vivo* and impaired relaxation in aortic rings in response to acetylcholine *in vitro* [143]. Lu et al. [142] also found that platelet and endothelial MPs positively correlated with markers of endothelial injury and platelet activation (von Willebrand factor and p-selectin, respectively) in patients with CKD [142]. Previously, Dursun et al. [145] studied the association between endothelial MPs (CD144^+^), arterial stiffness, and atherosclerosis in children suffering from CKD. The authors reported that the numbers of plasma endothelial MPs were higher in dialysis patients in comparison to predialysis patients and healthy volunteers. Furthermore, it was found that endothelial MPs positively correlated with blood pressure, C-reactive protein (CRP), parathyroid hormone, and disease duration, while they correlated negatively with glomerular filtration rate (GFR) and albumin levels [145]. Therefore, we can understand that MPs are active players in endothelial dysfunction that can later progress to cardiovascular complications and are not only biomarkers of endothelial cell activation and injury.

MPs themselves can be used to monitor response to treatments. The antidiabetic agent teneligliptin and the lipid-lowering agent simvastatin were tested for their effect in protecting against endothelial dysfunction in patients with diabetic nephropathy to prevent the progression to cardiovascular disease seen in this population of patients. Since MPs are markers of and contributors to endothelial dysfunction, as discussed earlier, they were measured to monitor the protective effects of these medications. Teneligliptin strongly reduced the levels of plasma platelet-derived MPs and the plasminogen activator inhibitor, a hallmark of platelet activation, in diabetic patients receiving hemodialysis suggesting its protective effects in this population [146]. Likewise, the treatment of diabetic patients suffering from CKD with simvastatin resulted in a significant reduction of procoagulant MPs (PS positive), platelet MPs, and monocyte MPs, suggesting its beneficial effect in ameliorating endothelial dysfunction [147].

Altogether, MPs can serve as a diagnostic marker because they are noninvasive, predict early pathological changes in the endothelium, and can predict the risk of other diabetic complications. MPs can also be used for monitoring treatment effects. Finally, therapeutic agents can be developed to target MPs as they appear to have a role in the pathophysiology of diabetic nephropathy and other complications.

## 8. Future Directions

Multiple clinical and preclinical investigations assessed the impact of treatments of diabetes and its complications on plasma levels of MPs. Esposito et al. [148] evaluated the actions of two oral antidiabetics, pioglitazone and metformin, on the numbers of plasma MPs originating from endothelial cells and EPCs in newly diagnosed type-2 diabetes patients. This study found that following 24 weeks of treatment with pioglitazone, patients' plasma exhibited a decrease in endothelial MPs while the numbers of EPC-shed MPs increased [148]. Furthermore, type-2 diabetes patients who received miglitol, an oral antidiabetic drug, for a period of 4 months, had lower levels of MPs of platelet origin compared to controls [149]. Shimazu et al. [150] reported earlier that another orally available antidiabetic treatment, acarbose, also reduced circulating numbers of platelet-shed MPs in type-2 diabetes patients [150]. Similarly, teneligliptin, a dipeptidyl peptidase-4 (DPP-4) inhibitor, significantly reduced plasma levels of platelet-shed MPs and plasminogen activator inhibitor in type-2 diabetes patients receiving hemodialysis [146]. The use of statins was also reported to affect the circulating levels of MPs patients with diabetes. Almquist et al. reported that simvastatin significantly reduced total PS-positive MPs, platelet-derived MPs, and monocyte-derived MPs in patients with diabetes and CKD [147].

Approaches to manage body weight were also observed to modify circulating levels of MPs from select cellular origins. Morel et al. [151] reported that a very low-calorie diet for 90 days caused a reduction in body weight in obese women and lowered plasma levels of MPs from different origins including procoagulant, leukocyte, and lymphocyte MPs. Furthermore, the diet improved several cardiac and metabolic vitals such as leptin levels and blood pressure [151]. Moreover, endothelial-derived MP levels were found to be reduced following a high-intensity exercise in overweight inactive participants [152].

Treatments based on the use of natural nanoparticles were also found to be effective in reducing the number of plasma MPs. For instance, Garavelo et al. [153] reported recently that the treatment of a rabbit model of atherosclerosis, fed a high-cholesterol diet, with natural nanoparticles obtained from medicinal plants (PTC) combined with trans-Sialidase (TS) for 6 weeks, reduced the number of total MPs in addition to those positive for *Mycoplasma pneumoniae* and oxidized LDL. Furthermore, the treatment of animals with PTC and TS reduced atherosclerotic plaque area and caused a positive remodeling of ascending aortic segment [153]. These findings highlight the potential of nanoparticle-based therapies in modulating the circulating levels of MPs.

Since MPs are implicated in the onset and progression of cardiovascular pathologies and diabetes-induced complications, they can be targeted therapeutically by many strategies. One strategy is controlling their release to reduce their levels; however, currently, there is a need to understand the mechanisms involved in the production and shedding of MPs to better and specifically target them. Another strategy could be the inhibition of their uptake by modulating specific surface lipids of MPs to prevent deleterious messages from reaching the recipient cells or inhibiting MP's surface ligands that interact with cell surface receptors, in order to prevent cell signaling. Finally, the modulation of the cargo content of the MPs to control the biological messages that they carry can be another strategy.

## Figures and Tables

**Table 1:** Major differences between extracellular vesicles found in body fluids.

	Apoptotic bodies	Microparticles (MPs)	Exosomes
Size of vesicles	>1,000 nm	100 nm–1,000 nm	<100 nm
Isolation method	Low to moderate speed centrifugation (<10,000 *g*)	High-speed centrifugation (<21,000 *g*)	Ultracentrifugations (>100,000 *g*)
Origin	Cytoplasmic membrane	Cytoplasmic membrane	Endocytic lysosomal system
Mechanism of shedding and release	Cytoplasmic membrane cytoskeletal rearrangement and loss of membrane asymmetry and release of bodies	Cytoplasmic membrane cytoskeletal rearrangement and loss of membrane asymmetry and release of bodies facilitated by calcium-dependent degradation of cytoskeleton	Fusion of multivesicular bodies (MVBs) with cytosolic membrane and exocytosis to extracellular environment
Most used techniques for detection and quantification	Flow cytometry, immunofluorescence, electron and atomic force microscopies, light scattering technique, western blotting, ELISA-based tests	Flow cytometry, immunofluorescence, electron and atomic force microscopy, light scattering technique, western blotting, ELISA-based tests	Immunofluorescence, electron microscopy, light scattering technique, western blotting, ELISA-based tests
Commonly used markers for detection	High levels of phosphatidylserine	Selectins, integrins, cell-specific surface antigens (cluster of differentiation or CD), phosphatidylserine	Tetraspanins (e.g., TSPAN29 and TSPAN30), ESCRT components, PDCD6IP, TSG101, flotillin, MFGE8, lactadherin, LAMP1

Abbreviations: ESCRT, endosomal sorting complexes required for transport; LAMP1, lysosomal-associated membrane protein 1; MFGE8, milk fat globule-EGF factor 8 protein; PDCD6IP, programmed cell death 6-interacting protein; TSG101, tumor susceptibility gene 101; TSPAN, tetraspanins.

**Table 2:** Summary of the main studies reporting diabetes-induced complications associated with altered levels of circulating MPs in humans and *in vitro* and animal models.

Complications	Origins of MPs and markers used for detection	Cell and animal models	Humans	Key observations	References
Diabetic nephropathy	(i) Urinary podocyte MPs (annexin V^+^/podoplanin^+^)		Patients with uncomplicated type-1 diabetes	(i) Podocyte-derived MPs are higher in type-1 diabetes patients compared to healthy volunteers and increase under hyperglycemic clamp	[141]
	(i) Platelet MPs (AV^+^/CD41^+^) (ii) Endothelial MPs (AV^+^/CD62E^+^)		Chronic kidney disease (CKD) patients	(i) Positive correlation between platelet- and endothelial-derived MPs with p-selectin (marker of platelet activation) and vWF (marker of endothelial injury)	[142]
	(i) Endothelial MPs (CD144^+^)		End-stage renal failure (ESRD) patients	(i) Endothelial MPs correlated with vascular dysfucntion *in vivo* (loss of flow-mediated dilation, elevated aortic pulse wave velocity, and common carotid augmentation index) (ii) *Ex vivo*, endothelial MPs from ESRD patients impaired endothelium-dependent relaxation and cGMP generation in aortic rings from Wistar rats	[143]
	Endothelial MPs (CD31^+^/CD41^−^)		End-stage renal failure (ESRD) patients	(i) Endothelial MP levels are independent predictors of all-cause and cardiovascular mortality in ESRD patients	[154]
	Urinary podocyte MPs (podocalyxin^+^)	Mouse models of diabetic nephropathy		(i) Higher podocyte-derived MPs were observed in mouse models of diabetic nephropathy [streptozotocin- (STZ-) treated, OVE26, and Akita mice]	[140]
	(i) Annexin V^+^ MPs (ii) Platelet MPs (annexin V^+^/CD41^+^, CD41^+^/CD62P^+^, CD41^+^/CD142^+^, CD41^+^/CD154^+^) (iii) Endothelial (annexin V^+^/CD144^+^, annexin V^+^/CD62E^+^, CD62E^+^/CD142^+^) (iv) Monocyte MPs (annexin V^+^/CD14^+^, CD14^+^/CD142^+^)		Patients with diabetic nephropathy	(i) Apart from TF (CD142^+^)-positive MPs, all other subpopulations were higher in diabetics with CKD compared to diabetics without (ii) All MP subtypes correlated negatively with GFR	[147]
	(i) Endothelial MPs (CD144^+^ and CD146^+^)		Children with CKD	(i) Endothelial MPs were positively associated with blood pressure, age, disease duration, CRP, and parathyroid hormone and negatively associated with hemoglobin, GFR, and albumin (ii) Pulse wave velocity was independently related to endothelial MPs	[145]
	(i) Endothelial MPs (CD144^+^or CD146^+^) (ii) Platelet MPs (CD41^+^) (iii) Leukocyte MPs (CD45^+^) (iv) Annexin V^+^ MPs		Patients with chronic renal failure who were undialyzed (CRF) or hemodialyzed (HD)	(i) Endothelial MPs (CD144^+^ and CD146^+^) were higher in CRF and HD patients compared to nondiseased controls	[144]

Diabetic retinopathy	(i) Mesenchymal stem cell-derived extracellular vesicles (levels not measured) obtained in diabetes-mimicking conditions	Human retinal pericytes		(i) Mesenchymal stem cell-derived MPs stimulated retinal pericyte detachment and endothelial cell proliferation *in vitro*	[139]
	Vitreous MPs: (i) Annexin V^+^ MPs (ii) Platelet MPs (CD41^+^) (iii) Endothelial MPs (CD144^+^)		Patients with diabetic retinopathy	(i) MPs promoted angiogenesis *in vivo* (endothelial cell migration and new vessel formation)	[137]
	(i) Platelet MPs (CD62P^+^ and CD63^+^)		Patients with diabetic retinopathy	(i) Platelet MP count increased with the progression of retinopathy	[155]
	(i) Monocyte MPs (annexin V^+^/CD14^+^)		Patients with diabetic retinopathy	(i) Monocyte MP levels significantly correlated with levels of platelet activation markers (platelet-drived MPs, CD62P^+^, and CD63^+^) and adhesion molecules (p-selectin and ICAM-1)	[156]
	(i) Monocyte MPs (annexin V^+^/CD14^+^)		Patients with diabetes and diabetic complications (nephropathy, retinopathy, or neuropathy)	(i) Elevated levels of monocyte-derived MPs correlated positively with platelet activation markers (platelet MPs, CD62P^+^, and CD63^+^)	[62]

Ischemic diseases and diabetic cardiomyopathy	(i) Platelet MPs (CD62P^+^)	Rat model of streptozotocin- (STZ-) induced diabetes and cardiomyopathy		(i) MPs reduced endothelium-dependent relaxation and eNOS expression in carotid arteries	[86]
	(i) Endothelial MPs from apoptotic origin (annexin V^+^/CD31^+^) (ii) Activated endothelial MPs (CD62E^+^)		Metabolic syndrome patients with chronic heart failure	(i) Patients with chronic heart failure had higher numbers of annexin V^+^/CD31^+^ MPs and lower levels of CD62E^+^ MPs compared to patients without (ii) Biomarkers of biomechanical stress (NT-proBNP, OPG, and hs-CRP) were independent predictors of the decreased ratio of CD62E^+^ MPs to annexin V^+^/CD31^+^ MPs	[74]
	(i) Endothelial MPs (annexin V^+^/CD31^+^)		Stable coronary artery disease patients	(i) High levels of endothelial MPs were associated with a high risk of cardiovascular mortality and need for neurovascularization (ii) Endothelial MPs independently predicted future major adverse cardiovascular and cerebral events	[157]
	(i) Platelet MPs (CD61^+^, CD61^+^/CD62P^+^, CD61^+^/fibrinogen^+^, CD61^+^/TF^+^)		Diabetic patients with atherosclerotic disease (e.g., stroke, ischaemic heart disease, or peripheral arterial disease)	(i) Soluble plasma p-selectin was higher in these patients, but no significant correlation was found between the levels of platelet-derived MPs and soluble plasma p-selectin	[158]
	(i) Endothelial MPs (CD144^+^) (ii) Platelet MPs (CD41^+^)		Type-2 diabetes patients with coronary noncalcified plaques	(i) Endothelial- and platelet-derived MPs correlated with high levels of hs-CRP (ii) Endothelial MP numbers were higher in the presence of noncalcified cornonary diseased segments	[71]
	(i) Endothelial MPs (CD144^+^)		Patients with type-2 diabetes and coronary artery disease	(i) Significant association between elevated levels of endothelial MPs and endothelial injury *in vitro* and impaired endothelium-dependent vasodilation *in vivo* (ii) Elevated levels of endothelial MPs were a significant risk factor for coronary artery disease in type-2 diabetes patients	[70]
	(i) Apoptotic endothelial MPs (annexin V^+^/CD31^+^)		Coronary artery disease patients	(i) Elevated MP counts positively correlated with impaired cornonary endothelial-dependent vasodilation in coronary artery disease patients (ii) High levels of endothelial MPs were an independent predictor of the severity of endothelial dysfunction	[60]

Abbreviations: GFR, glomerular filtration rate; hs-CRP: high-sensitive C-reactive protein; ICAM-1, intracellular adhesion molecule-1; NT-proBNP, N-terminal pro b-type natriuretic peptide; OPG, osteoprotegerin; TF, tissue factor; vWF, von Willebrand Factor.
